# New techniques for motion-artifact-free *in vivo* cardiac microscopy

**DOI:** 10.3389/fphys.2015.00147

**Published:** 2015-05-12

**Authors:** Claudio Vinegoni, Sungon Lee, Aaron D. Aguirre, Ralph Weissleder

**Affiliations:** ^1^Center for Systems Biology, Massachusetts General Hospital and Harvard Medical SchoolBoston, MA, USA; ^2^School of Electrical Engineering, Hanyang UniversityAnsan, South Korea

**Keywords:** cardiac imaging, motion artifacts, laser scanning microscopy, motion compensation, image stabilization

## Abstract

Intravital imaging microscopy (i.e., imaging in live animals at microscopic resolution) has become an indispensable tool for studying the cellular micro-dynamics in cancer, immunology and neurobiology. High spatial and temporal resolution, combined with large penetration depth and multi-reporter visualization capability make fluorescence intravital microscopy compelling for heart imaging. However, tissue motion caused by cardiac contraction and respiration critically limits its use. As a result, *in vitro* cell preparations or non-contracting explanted heart models are more commonly employed. Unfortunately, these approaches fall short of understanding the more complex host physiology that may be dynamic and occur over longer periods of time. In this review, we report on novel technologies, which have been recently developed by our group and others, aimed at overcoming motion-induced artifacts and capable of providing *in vivo* subcellular resolution imaging in the beating mouse heart. The methods are based on mechanical stabilization, image processing algorithms, gated/triggered acquisition schemes or a combination of both. We expect that in the immediate future all these methodologies will have considerable applications in expanding our understanding of the cardiac biology, elucidating cardiomyocyte function and interactions within the organism *in vivo*, and ultimately improving the treatment of cardiac diseases.

## Introduction

Despite recent improvements in health care, cardiovascular diseases are still responsible for a stunning 30% of deaths worldwide (Go et al., 2013). Among them, myocardial infarction (MI) is the most frequent cause. In patients with MI, ischemia is commonly caused by atherothrombotic occlusion of a coronary artery, triggered by sustained local and systemic inflammatory response. Rapid neutrophil accumulation occurs first, followed by monocytes, which dominate the cellular infiltration in the infarcted area for several days regulating the evolution of heart failure (Chien et al., 2008; Nahrendorf et al., 2010; Woollard and Geissmann, 2010). Mouse models of myocardial infarction and heart failure have offered clinically relevant mammalian systems for the investigation of fundamental biological processes. Understanding the dynamics of the key cellular mediators of injury and healing in the heart may offer important new approaches to MI treatment and prevention of subsequent heart failure.

So far, the knowledge of these processes has been hampered by the absence of high resolution imaging techniques. While modalities such as MRI, ultrasound, and PET can provide information about macroscopic whole organ function, they lack the necessary resolution to give insight into single cell biology and physiology. Intravital confocal and multiphoton microscopy imaging has provided profound insights into *in vivo* cell biology (Pittet and Weissleder, 2011; Ritsma et al., 2012) offering high spatial and temporal resolution as well as deep-penetration depth and multi-reporter visualization. These capabilities have in turn enabled the acquisition of cellular information under natural physiological conditions and offered unique opportunities to explore and investigate biology in living systems.

At present, the vast majority of **intravital microscopy** imaging set-ups rely on skinfold window chambers (Lehr et al., 1993) or organ exteriorization. Unfortunately these approaches are not suitable for all organs, particularly the heart. Imaging at orthotopic locations is therefore preferable and often necessary.

**Figure 1:**
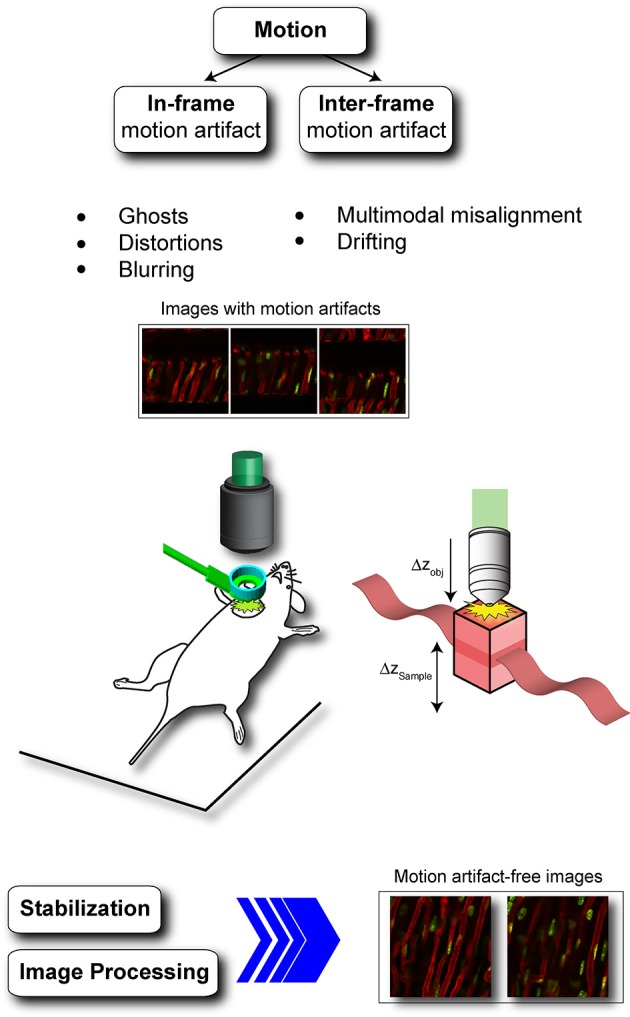
**Physiological movements induce motion artifacts in acquired images**. In-frame and inter-frame are the two most common types of motion artifacts. In-frame motion artifacts refer to image degradation present within a single image and include ghosts, distortions and blurring while inter-frame motion artifacts refers to motion between consecutive frames due for example to multimodal misalignment and/or animal or imaging probe drifting. During *in vivo* heart imaging both classes of artifacts are present, making impossible to visualize the heart without the adoption of proper motion stabilization methods. Adapted from Lee et al. (2014).

Until now, attempts to image organs *in vivo* within the body have been severely hampered by **motion-induced artifacts**, the removal of which has remained an ongoing challenge (Figure 1). Generally, both cardiovascular and respiratory movements tend to propagate throughout the body, modulating in time the position of every organ. While several motion suppression techniques have been developed, their use has been largely restricted to organs that move less and more slowly. In particular *in vivo* imaging of the beating heart has been quite problematic due to its inherent rapid contractility and great displacement in motion. For all these reasons, most studies so far have relied on non-contracting Langendorf heart preparations or transplanted models critically limiting our understanding of the heart's natural physiology and function in the living body.

**Figure 2:**
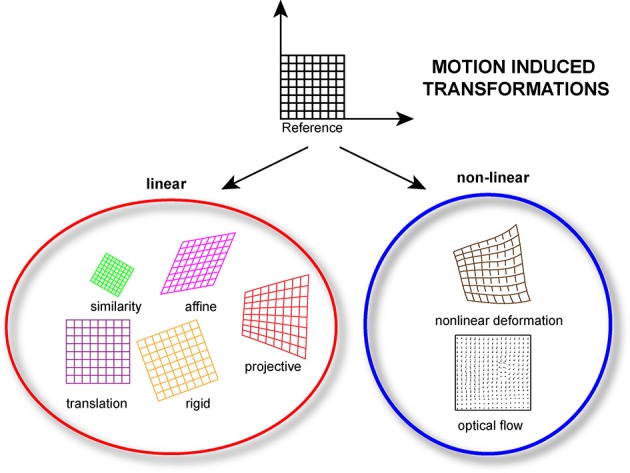
**Linear and/or nonlinear transformation models can be implemented during the post-processing phase of the acquired data**. Linear models include translation, rigid (translation + rotation), similarity (translation + rotation + scale), affine and projective transformations. Nonlinear models, which consider non-linear transformations allow for more complex deformations.

Motion-induced imaging artifacts are inherent in the acquisition nature present in laser scanning microscopy (LSM), where a sampling point scans over time different points within the field of view, and they can be generally classified in in-frame and inter-frame motion distortions (Figure 2).

High-speed imaging (100 frames per second) in combination with simple frame rejection is very effective in suppressing these effects, but acquisition at this speed is not always feasible for *in vivo* imaging due to poor signal to noise ratio (spinning disk microscopy) or extremely limited penetration depth (CCD imaging). Alternative solutions have been proposed lately with several examples present in the literature. Here, we report results from our recent work and from others focused specifically on compensation of motion artifacts for high resolution imaging of the beating heart *in vivo*. In particular we introduce the concept of **gated “sequential segmented microscopy”** (SSM), passive and active stabilization schemes, and image-processing algorithms for automatic motion-artifacts removal.

## Stabilization methodologies

Several stabilization methodologies have been developed for *in vivo* organ imaging, and these techniques differ in approach and complexity depending on the particular organ of interest. Here we illustrate different approaches we have recently used for cardiovascular applications.

### Passive stabilizers to compensate motion

The most straightforward way to remove, limit or confine, an organ's motion is to physically immobilize it. This can be typically achieved with the use of a rigid support by introducing mechanical restriction and tight confinement of the imaged tissue. Its implementation occurs in several configurations for example through window chambers (Kedrin et al., 2008; Holtmaat et al., 2009; Farrar et al., 2012; Ritsma et al., 2013), or by way of a compressive cover slip. The latest approach is immediate in its use and very effective in providing motion amplitude reduction. Unfortunately these constraints have a negative impact when used in the heart and can impair physiological functions or lead to permanent damage. The only instance in which this methodology appears to work reasonably well is in transplanted hearts as demonstrated by Li et al. who managed to image a beating mouse heart transplanted into the right cervical region of a recipient mouse (Li et al., 2012). Here, after partial exteriorization, the heart was stabilized using the simple pressure of a glass coverslip of an upper chamber plate.

**Figure 3:**
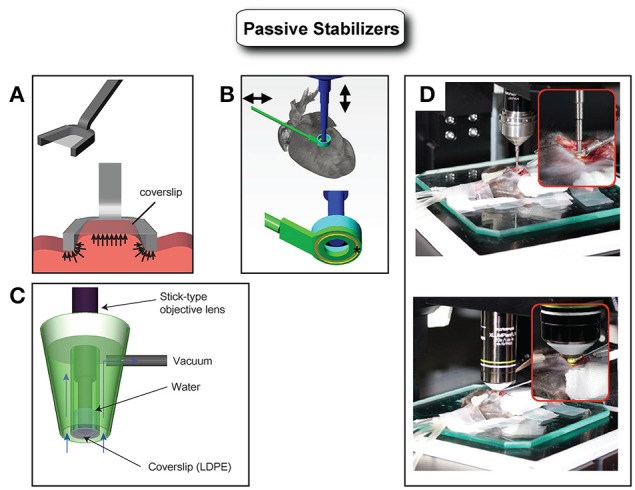
**Various types of passive stabilizers. (A)** Holding stabilizers consist generally of an arm and a coverslip. The arm constrains the horizontal movements while the coverslip limits the vertical component of the motion. This type of stabilizer (compressive) has been demonstrated with good results in particular for kidney or liver imaging. However, it is not suitable for heart imaging because heart movement is very large in displacement and mechanically stiff. The strong pressure that would be required for heart immobilization using this type of stabilizer would easily affect the physiology leading to heart arrest. Adapted from Lee et al. (2012b). **(B)** Adhesive based mechanical stabilizers can immobilize the mouse heart sufficiently without relying on strong pressure application. Adapted from Lee et al. (2012a). **(C)** Alternatively, a suctioning mechanical stabilizer based on mild negative pressure has been shown to immobilize the beating mouse heart with optimal motion artifacts suppression. Adapted from Vinegoni et al. (2012). **(D)** Images of intravital imaging setups using the stabilizer as shown in B.

To overcome the limitations imposed by an approach based on physical immobilization, various stabilizers have been proposed (Figure 3). Specifically small-sized mechanical holders, which are easy to be positioned inside the abdomen, have been demonstrated by several groups (Cao et al., 2012; Lee et al., 2012b). However, the fast and strong cardiac motion components don't allow for optimal artifacts removal and prevent high resolution imaging.

Very recently, several researchers have effectively overcome these difficulties with the design of a new class of stabilizers utilized in combination with novel acquisition schemes. The idea behind these methods relies on the fact that motion compensation can be achieved even in the presence of strong motion components, providing reproducibility in position is present over time. Here the stabilizers are used to mitigate in part the motion components but more importantly to introduce spatial reproducibility along the physiological cycles. By adjusting the timing of the acquisitions, artifact-free images can then be reconstructed as better illustrated in the next section. Following this approach high resolution *in vivo* imaging of the beating heart has been successfully obtained by our group by combining a novel adhesive stabilizer with a gating acquisition algorithm (Lee et al., 2012a). Here, a custom-designed stabilizer consisting of a long rigid metal shaft and a flat ring at one end is bonded to the myocardium with a thin layer of approximately 50 μm of Dermabond, a clinical grade bonding agent commonly employed in cardiac surgery and FDA approved for human surgical use.

Other solutions based on a water-immersion suctioning stabilizer have been proposed as well. Unlike compressive stabilizers where positive pressure is present, here the holder does not restrict the natural blood flow through the myocardium. Instead, a slight negative pressure allows for the heart to maintain its original position within the chest cavity, while stabilization occurs. In addition, the suctioning stabilizer offers the possibility of sampling multiple areas during a single imaging sessions (Vinegoni et al., 2012). Similarly, other groups used a suction-assisted endoscope integrated into a confocal microscope for imaging the beating heart through an incision. A suction tube covering the endoscope stabilizes the local motion of the imaged tissue (Jung et al., 2013).

In addition to the passive stabilizers mentioned above alternative designs are expected to be proposed in the near future and to be extensively used for *in vivo* cardiac studies in the mouse.

### Gated sequential segmented microscopy (SSM) for artifact-free high resolution imaging

Mechanical stabilization approaches based on suctioning or bonding can be quite efficient at reducing the heart's range of motion by several orders of magnitude while allowing for blood flow and minimal physiological perturbations. However, they generally do not completely eliminate all physiological movements. This can be ascribed to the cardiac contractions which are taking place during the microscope acquisition time, with one beat occurring approximately every 120 ms. When considering also respiratory activity, the two effects add together making it particularly troublesome when aiming for high resolution imaging, with acquisition times which are in general comparable to, or longer than, one ECG cycle. To overcome this issue, novel triggering schemes based both on cardiac and respiratory gating need to be implemented in combination with the use of a stabilizer.

**Figure 4:**
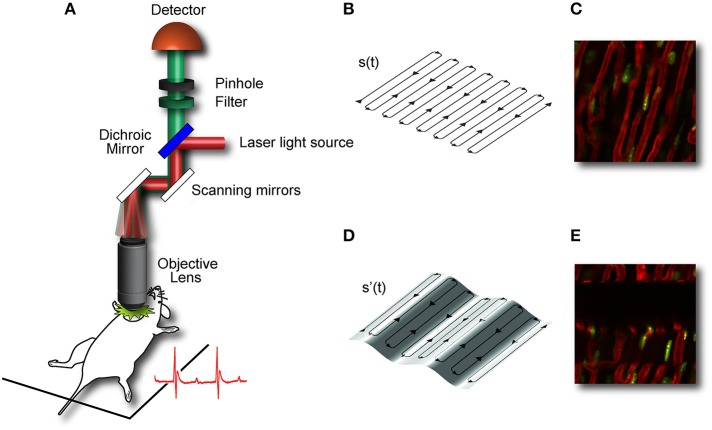
**Image acquisition in laser scanning microscopy (LSM) is based on sequential point-by-point excitation**. Fluorescence emission is detected in epi-collection along a scanned **(A)** or de-scanned detection path, depending if confocal or multiphoton microscopy is chosen. The collection of the signal in correspondence of each point along the scanning path gives rise to an image. When the imaged subject is stationary, the excitation scanning point draws a pre-defined trajectory across a horizontal imaging plane perpendicular to the objective optical axis. The trajectory will then represent a planar section of the imaged organ **(B,C)**. When the imaging subject is in motion (here for simplicity we assume only vertical motion is present) the scanning point in the reference frame of the organ, does not describe a plane but instead a curved surface whose profile is modulated in time by the physiological components of the motion **(D,E)**. Adapted from Lee et al. (2012b).

During laser scanning microscopy imaging, the excitation scanning point draws a continuous pre-defined trajectory (“raster path”) across a horizontal imaging plane, which is a periodic function of time and determined by the dwelling time, the number of pixels per line and the total number of lines per image (Figure 4A). This surface is equivalent to a horizontal plane with respect to the laboratory frame of reference (Figures 4B,C). But due to the presence of physiological-induced motion components, all points belonging to the raster path will not lie at the same depth within the imaged organ. Instead in the frame of reference of the organ, the imaging plane will be equal to a surface modulated in time by both breathing and cardiac frequency as well the acquisition imaging parameters (Figures 4D,E). Therefore, the acquired image is not representative of a horizontal plane intersecting the organ of interest, but instead it will resemble a curved one. This implies in-frame motion artifacts such as distortion and blurring will intrinsically affect the images with a severity which varies, during the physiological cycle, in relation to the organ's instant speed.

The problem of motion compensation in acquired data has been a subject of intensive study in different imaging modalities and several motion reduction methods have been developed for magnetic resonance imaging (MRI), particularly for high resolution cardiac MRI (Wiesmann et al., 2003; Scott et al., 2009). While the basic imaging fundamentals of MRI and optical microscopy are distinctively different, the underlying principles for image stabilization can be translated in both techniques and analogies can be drawn between MRI compensation schemes and LSM imaging protocols.

**Figure 5:**
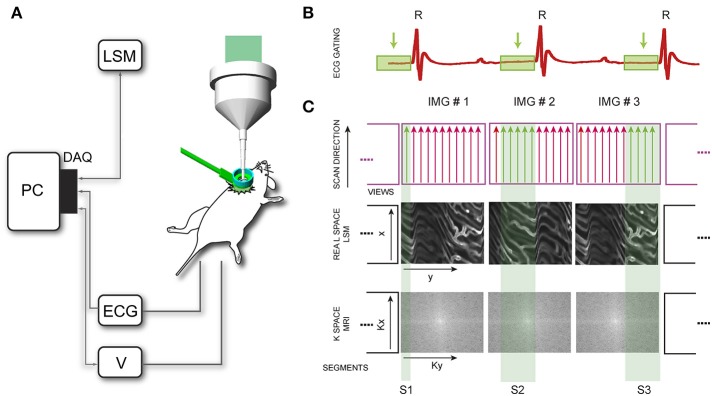
**Scheme of principle for motion compensation in laser scanning microscopy (LSM). (A)** DAQ, data acquisition card; ECG, electro-cardiogram; V, mechanical ventilator. **(B)** Time-gated windows, coincident with the time window corresponding to the end-diastole, are isolated in the recorded ECG. **(C)** In LSM images are acquired pixel by pixel in the real space, with the excitation scanning laser beam moving along parallel lines (MRI analog of a segment or group of views). Segments are then sequentially collected within the time-gated window identified in **(B)** until the entire real space is filled. In a typical high resolution cardiac MRI imaging session instead the sequence of views is collected in the k-space, by varying the phase encoding gradient. Adapted from Vinegoni et al. (2013).

“Segmented cardiac-gated acquisition” (Figure 5) is a measurement protocol commonly used in cardiac MRI for acquisition of images corresponding to a specific phase of the cardiac cycle and used to correct for cardiac motion artifacts. In this protocol only data belonging to specific time windows in the MRI k-space are selected and then combined to fill the entire space in a “line-by-line” or “view per view” acquisition fashion. A full k-space MRI image can then be reconstructed by considering all image “segments” (i.e., ensemble of adjacent views) that correspond to different times points of a cardiac phase. Two schemes are commonly utilized for data acquisition, which are based on active or passive processing conditions also referred as “retrospective gating,” or “prospective triggering.” In the first modality different parameters such as the ECG signal or the mechanical ventilator pressure profile are simultaneously collected together with the raw image sequences, with different lines of the k-space individually and sequentially acquired. The lines belonging to a precise gating window T_GW_ of the cardiac cycle, are then gated out from the raw images, and merged to obtain a final image lacking motion artifacts and representative of the imaged organ at a determined physiological cycle. If a time delay between T_GW_ and the gating signal is properly chosen and progressively incremented, all k-space segments, which are representative of a cardiac phase are combined and an artifact-free image is then obtained. Another possible scheme based on “prospective triggering” acts in a similar way as the former one, with the only difference that acquisition is started only during specific time points within the ECG, and identification of the cardiac and respiratory phases is based on real time data analysis. The advantage of this gating scheme over the other relies on the fact that acquisitions are performed only for a reduced amount of time and no un-necessary data is collected. Therefore, the number of raw images necessary to build a reconstructed image, are typically less in prospective acquisition mode in comparison to a retrospective approach.

**Figure 6:**
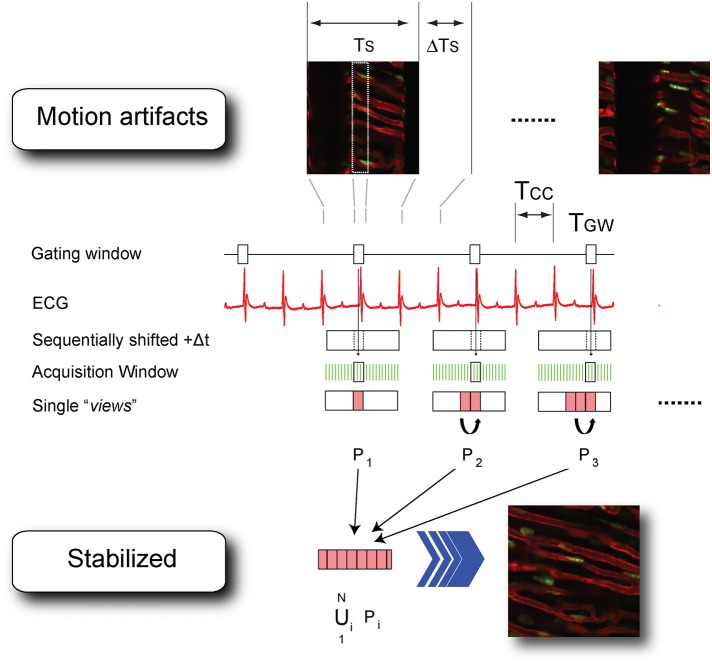
**Scheme of principle and timing diagram for retrospectively cardiac-gated sequential segmented laser scanning microscopy**. T_CC_, time interval between two cardiac cycles; TS time to acquire a single raw image; ΔTs the time interval between the end and the beginning of two consecutive acquisitions; T_GW_, time-gated window corresponding to the end-diastole. Segments P_i_ (i = 1…N) are isolated from each image in correspondence to the time-gated window T_GW_ and combined together in a final stabilized image which is truly representative of the heart's morphology at the cardiac phase corresponding to the time-gated window. Adapted from Vinegoni et al. (2013).

Assisted motion-synchronized scanning can be analogously extended in LSM, both in confocal or multiphoton mode (Vinegoni et al., 2013). In this approach, named here “sequential segmented microscopy” (SSM), horizontal image lines (“views”), described by the raster path of the excitation beam, move along the objective field of view and are isolated and grouped together in single patches (“segments”) which are gated out from sequentially acquired images. By choosing a convenient gating window T_GW_, coincident with the end diastole, minimal border discontinuities are present between the different segments giving rise to an image truly representative of a horizontal physical plane virtually slicing through the imaged organ (Figure 6).

**Figure 7:**
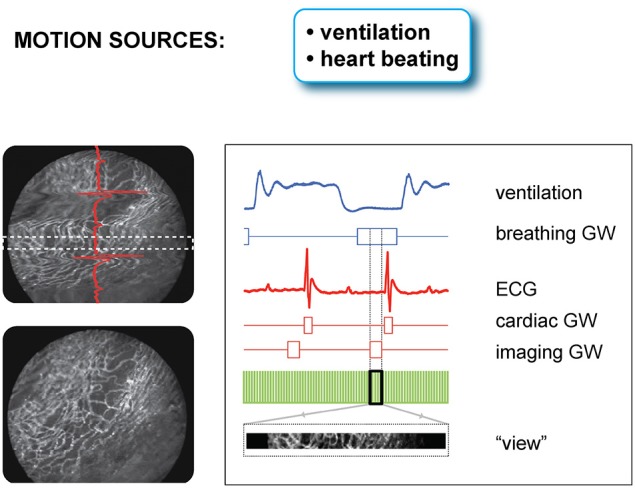
**Scheme of principle and timing diagram for retrospectively double gated (cardiac and respiratory) sequential segmented laser scanning microscopy**. Due to the combined effect of cardiac and respiratory motion, segments from raw images need to be chosen in correspondence to a time-gated window, which is the intersection of two distinct temporal windows present in the ECG and the ventilator pressure diagram. Adapted from Lee et al. (2012a).

Because respiration is typically controlled via mechanical ventilation in a pressure-cycle mode, image acquisition can be in a similar way gated to a particular period of the respiratory cycle located at a time window near the end of the inspiration or expiration phase. As a new gating window we need therefore to consider the intersection between the two cardiac and respiratory gating windows (Figure 7). This time interval corresponds to a simultaneous temporal period in which the motion of all organs is at a minimum, and during which all corresponding “segments” are representative of a unique horizontal plane as defined in the frame of reference of the heart. The number of images, which is necessary to collect in order to obtain a sufficient amount of segments to fill the real-space, is directly dependent on the microscopy acquisition parameters, such as the number of lines, the pixel per lines, and the dwell time, as well as their relation to the physiological attributes.

**Figure 8:**
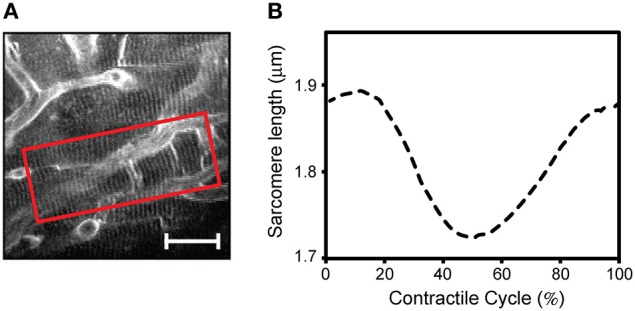
**Intravital two photon microscopy of cardiomyocyte function and structure in the beating heart. (A)** Two-photon microscopy imaging reveals subcellular structures in individual cardiomyocytes in the contracting heart. Using prospective sequential segmented microscopy, motion-artifact-free images can be formed at every point in the cardiac cycle, enabling visualization of myocytes' contractions. **(B)** Measurement of a single myocyte contractile cycle. The mean sarcomere length is calculated over the region of interest indicated in the red box in A, at every point in the cardiac cycle by Fourier transform analysis of the sarcomere striations. Adapted from Aguirre et al. (2014). (Scale bar; 20 μm.).

Prospective gating can be performed for intravital microscopy of the heart by precisely synchronizing the ECG with image acquisition. This could be done by triggering the microscope on the native ECG. Variability in heart rate, whether due to physiologic variation or due to abnormal heart rhythms, can be difficult to track, since most scanning microscopes are not capable of separately triggering sequential frames. A more robust solution takes advantage of the ability to pace the heart in intravital imaging preparations. Using the microscope acquisition signal to drive a pacemaker enables very accurate synchronization of image acquisition with the cardiac cycle (Aguirre et al., 2014). The contractile motion of the heart can be completely removed from the images using this method, and by shifting the phase of the acquisition relative to the paced ECG, all phases of the cardiac cycle can be sampled. This method of prospective sequential segmented microscopy (PSSM) enables motion-artifact free imaging throughout the cardiac cycle (Figure 8A) and allows measurement of contractile function of individual cardiomyocytes (Figure 8B).

It is important to underline the concept that to enable assisted motion-synchronized scanning, reproducibility over time in the position of the moving organs needs to be present at specific time gating windows within the physiological cycles. If this condition is satisfied, different segments obtained at different time points can be merged together and gated SSM is a viable methodology. This is exactly the purpose served by the mechanical stabilizers, which in addition to reduce the motion amplitude, introduce cyclic high spatial reproducibility (less than a micron) over the physiological periods.

### Active motion compensation for *in vivo* cell tracking

Although mechanical passive stabilizers can provide practical and efficient solutions for high resolution imaging in the beating heart, it is difficult to image the same physical plane over the entire cardiac and respiratory cycle. In SSM data are segmented at different time windows along the physiological cycles, and the reconstructed images will be representative of horizontal planes optically sectioning the heart at the respective gated windows. Because complete physical immobilization of the heart is impossible without negatively impacting its physiological functions and causing irreversible damages (e.g., vasculature rupture), residual axial motion will always be present. It is important to note that these axial motion components will not be detrimental in terms of planar image reconstruction. Instead they will manifest themselves in discontinuities alongside the axial direction and prevent us from imaging the same plane (with respect to the heart's frame of reference) over the different phases of the respiratory and cardiac cycles. This could be crucial to perform *in vivo* high resolution or when following the contraction of a single myocyte.

**Figure 9:**
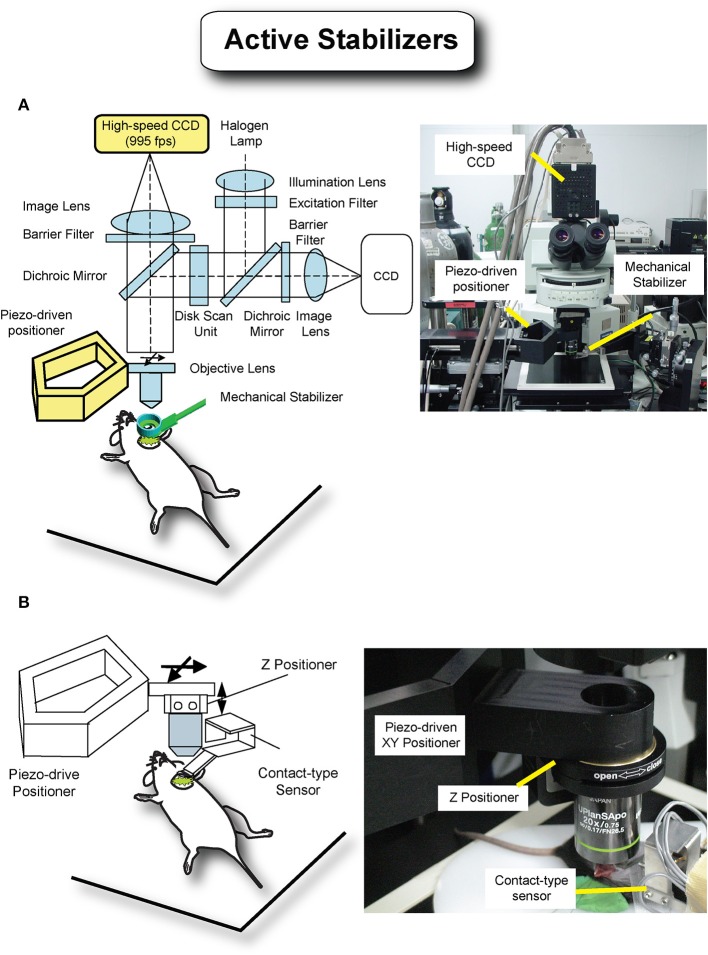
**Active motion stabilization removes relative movement between the imaging device and the imaged tissue by active motion of the objective lens and tracking of the imaged tissue, leading to motion-free images. (A)** A high-speed camera with 955 frames per second was utilized to track the movement of the tissue, and a piezoactuator-driven positioner was designed for precise and fast movement of the objective lens. Adapted from Lee et al. (2008). **(B)** A contact-type sensor consisting of three cantilevers beams with strain gauges was designed to measure the three dimensional movement of the tissue instead of the previous high-speed camera. This sensor also works as a passive stabilizer, reducing the movement with soft pressure.

One possible way to achieve axial stabilization for all phases of the cardiac cycle is through active motion compensation. In this method the relative residual motion present between the imaging device (i.e., objective) and the imaged tissue can be actively canceled by tracking in real time the tissue position (Nakamura et al., 2001; Lee et al., 2008; Yuen et al., 2009; Bakalar et al., 2012) and accordingly shifting the objective. This cancelation of relative movement virtually freezes in space both the objective and imaged sample leading to motion artifact free images. Several robotic motion compensation modalities based on active compensation schemes (Figure 9) have been demonstrated. Lee et al. (2008) recently implemented a high-speed visual feedback system demonstrating optimal compensation for respiration-induced movement (Figure 9A). Here a high-speed camera collects 2D images at a high frame rate measuring the motion of the stabilized organ. A piezo actuator-driven robotic closed arm, consisting of a five-bar linkage mechanism, moves the objective lens rapidly following the moving organ as determined by the camera. With this setup a maximum motion compensation in excess of 150 microns was obtained for a mouse under anesthesia and without ventilation. Other possible solutions can be based on a contact-type displacement sensor as a tracking device instead of the high-speed camera (Figure 9B). Information from the three strain gauges is used to estimate the displacement and track the tissue positions over time.

### Automatic motion artifact removal

In sequential segmented microscopy motion compensation is obtained combining raw acquisition with supplemental information. Typical examples as discussed above are the presence of a high-speed multichannel data acquisition system, a differential signal amplifier, a mechanical animal ventilation apparatus or a respiration sensor, in combination with the specific supporting functions from the imaging microscope such as the image and line triggering signals or the scanning timing output functions.

Because it is not always possible or convenient to obtain such additional data, alternative methods purely relying on **image processing algorithms** have been proposed. Planar in-frame motion distortions have been effectively compensated using motion distortion models based on constant velocity assumptions (Vercauteren et al., 2006). Inter-frame motion artifacts have been also corrected by image registration with realignment of the video-frame sequence. So far however distortion-free images had to be collected prior to imaging making the method (Liebling et al., 2005; Soulet et al., 2013), not applicable for heart imaging. Another methodology we have recently introduced, which does not require any a priori information or any knowledge about the animal physiology, has been successfully implemented for high resolution imaging of the heart *in vivo* (Lee et al., 2014). Here, motion-induced artifacts in the raw images are removed automatically through image processing utilizing the undergoing periodic characteristics of the physiological motions (introduced through passive stabilization and mechanical ventilation).

**Figure 10:**
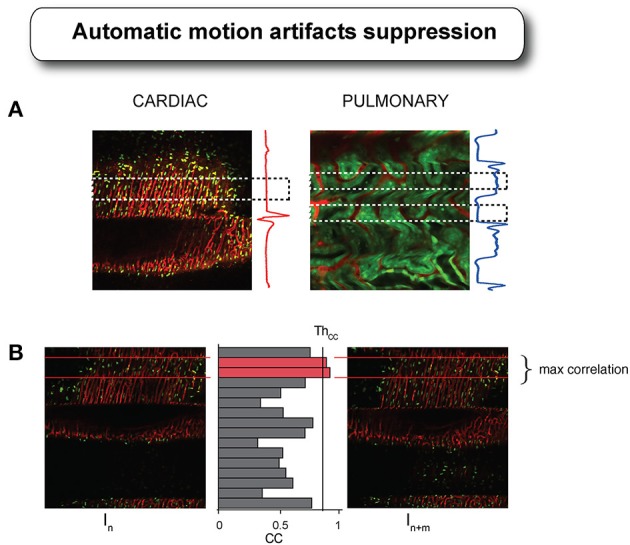
**Cardiac and respiratory motion artifacts present in the heart and in the kidney during laser scanning microscopy acquisition with their corresponding ECG and ventilator pressure traces. (A)** Dashed boxes indicate the temporal areas where no motion is present or minimal within the acquired images. **(B)** If reproducibility is present in certain part of the motion, as guaranteed by the presence of the tissue stabilizer, then images acquired at different time points will present “locally” different values of correlation. By combining all the segments with high values of correlation coefficient, it is possible to reconstruct a final motion artifact-free image. Adapted from Lee et al. (2014).

**Figure 11:**
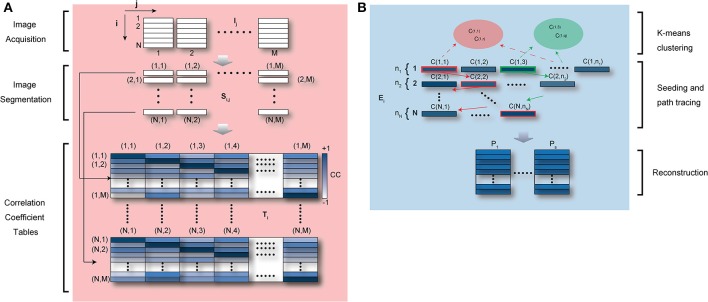
**Schematic diagram of automatic artifact removal and image reconstruction algorithm. (A)** M raw images, which include in-frame motion artifacts “locally” or “globally,” are divided in N segments. All M segments corresponding to the same temporal position in the image coordinates are collected and the correlation coefficient is calculated between each segment and all the remaining ones. N “segment correlation coefficient tables” are then obtained for each individual segment. In the table, dark color represents high values of correlation coefficient, i.e., similarity within segments. Adapted from Lee et al. (2014). **(B)** For each “segment correlation coefficient table,” all segments with a cross correlation higher than a set threshold are collected giving rise to a “similarity ensemble” E_i_. If the physiological cycle has multiple point of high stability (e.g., acquisition during ventilation), segments within the first “similarity ensemble” E_1_ may be classified into multiple clusters (the same number of clusters as stable points) using K-means clustering, and then used as seed for automatic image reconstructions (Lee et al., 2014).

Image acquisition in LSM is based on sequential point-by-point excitation (Figure 4) and it typically occurs within a time of approximately or larger than 100 ms. This time scale is of the order of one cardiac cycle making it feasible to find for each image an area with minimum cardiac movement in coincidence with the diastolic phase T_C_ (Figure 10A). An automatic artifact removal method can therefore be implemented to “automatically” identify all artifact-free segments in a sequence of images and stitch them together into a final motion-compensated image. The basic principle is illustrated in Figure 10B. Two images of a beating mouse heart are presented both showing significant motion artifacts. Despite their differences, we can quantify the similarity between the different segments of the images by building a correlation coefficient (CC) table of all the temporally overlapping segments. A CC bar graph, representing CC values for all individual segments, will present high values in correspondence of the motion-free areas indicated by the red lines in Figure 10B, while the other motion-distorted segments have low values of CC (high distortion). If we obtain a set of CC tables for a generic image sequence it is then possible to determine all motionless segments, to combine them according to their time coordinates and to reconstruct motion artifact-free image. This operation can be performed automatically through the use of an algorithm as proposed in Lee et al. (2014) and shown in Figure 11.

## Applications in cardiac imaging

**Figure 12:**
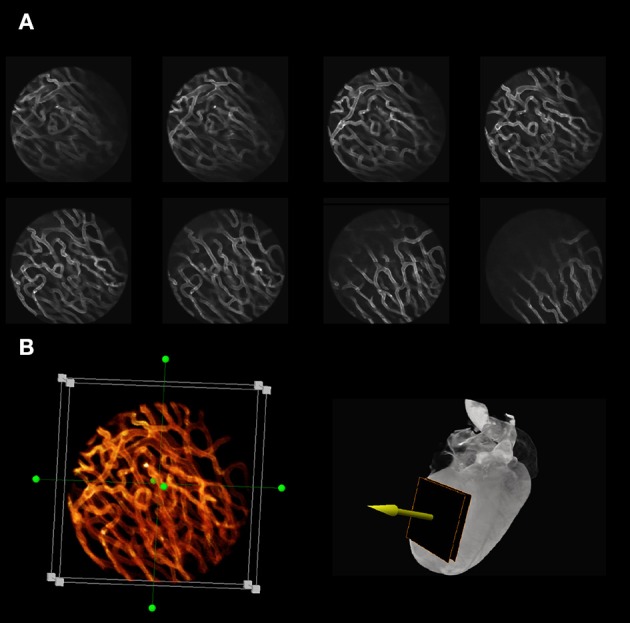
**Optical sectioning of cardiac microvasculature stained with lectin, obtained *in vivo* in the beating heart, after motion artifacts removal. (A)** Sequence of images taken at different depths in 5-μm increments using a ×20 MicroProbe objective. In-frame motion artifacts present within the raw images are removed and motion-free images are reconstructed using a retrospective gating scheme and sequential segmented microscopy (SSM). **(B)** Optical sectioning along the indicated direction (yellow arrow) allows for three-dimensional reconstructions of the beating heart *in vivo*.

**Figure 13:**
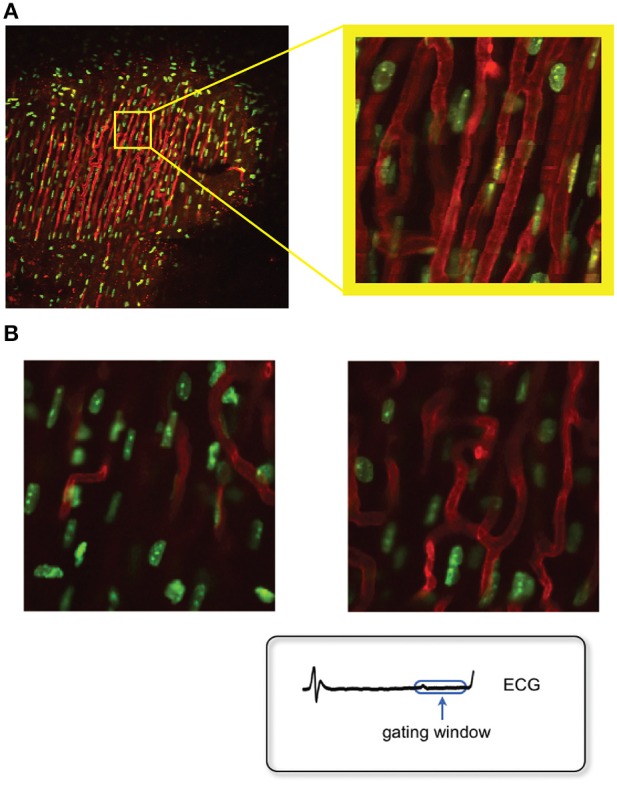
**Fluorescence images of the myocyte's nuclei and the microvasculature as measured in the beating heart *in vivo*, after motion artifact removal. (A)** Sequential segmented microscopy (SSM) allows for stabilized heart imaging enabling both large and small field of view imaging. Green: fluorescent Hoechst 33258 for nuclei staining; red: fluorescent lectin for staining of the myocardium capillaries. Adapted from Vinegoni et al. (2013). **(B)** High resolution stabilized images of the myocytes nuclei at different depths within the cardiac muscle can be obtained by optical sectioning. At high resolution imaging, both subcellular and subnuclear structures can be clearly resolved. Adapted from Lee et al. (2012a).

**Figure 14:**
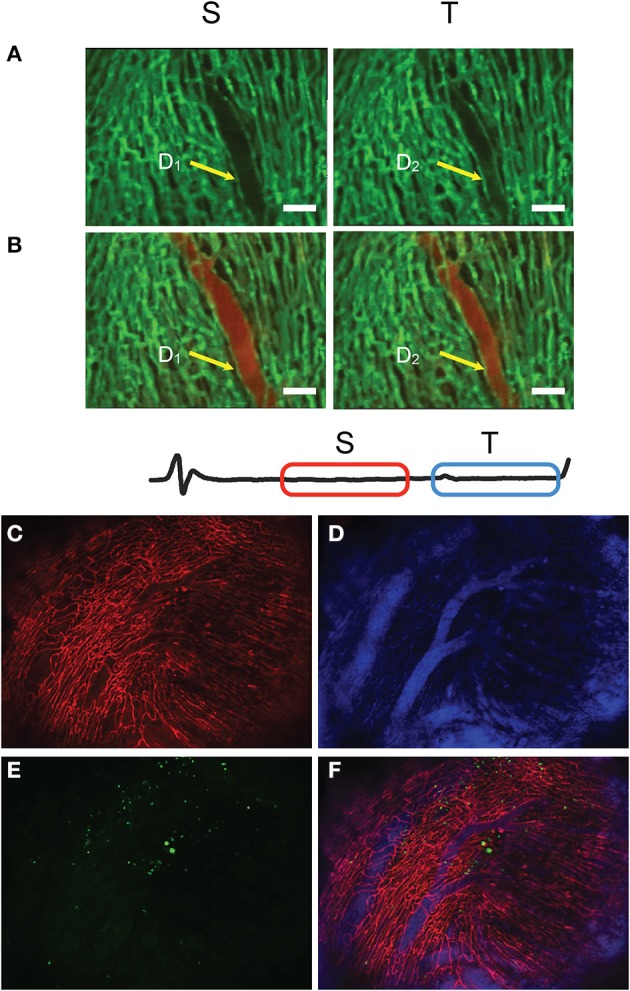
**Time-lapse fluorescence imaging of heart perfusion using a vascular pool imaging agent injected via tail vain**. Frames S and T are taken at two different time-gating windows within the ECG, corresponding to two distinct phases of the cardiac cycle. **(A)** Images taken before perfusion. **(B)** Images taken after perfusion. Changes in the vessel diameter are evident for the two phases. Green: fluorescent lectin staining of capillaries; red: fluorescent signal from the vascular pool agent Angiosense-680. **(C–F)** Large field of view images obtained with SSM of a heart which sustained an ischemia reperfusion injury before being injected with GFP-expressing cells, freshly isolated from the bone marrow of a donor mice. **(C)** Red represents the fluorescence lectin signal from stained capillaries; **(D)** blue the fluorescence signal produced by the blood pool imaging agent Angiosense-680; **(E)** green the GFP-expressing cells; **(F)** fusion of three images. Adapted from Lee et al. (2012a).

**Figure 15:**
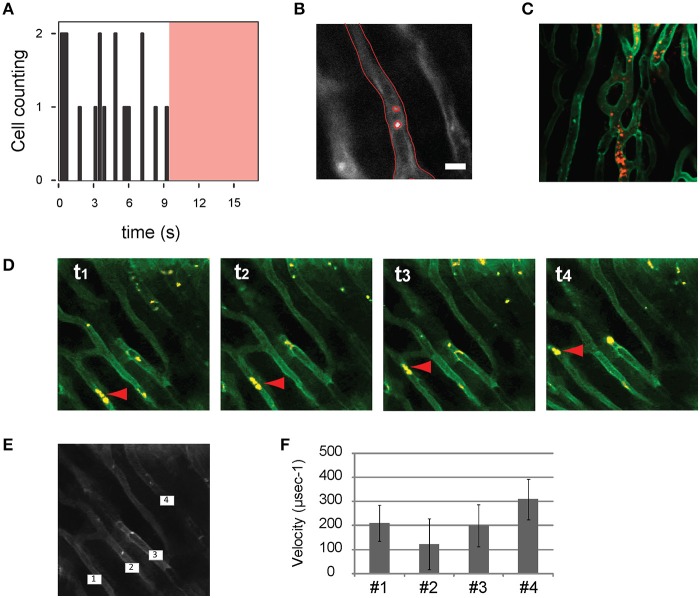
***In vivo* flow cytometry and cell tracking in the beating heart**. Images were acquired at 15 frames per second. **(A)** Number of leukocytes passing through the imaged capillary as a function of time within the region of interest indicated with a red border in **(B)**. In **(A)** the red shaded area indicates the time during which no leukocytes were transiting through the vessel. **(B)** Segmentation was performed to delineate the border of the vessel and the leukocytes. Leukocytes were stained with Rhodamine 6G (Ex 520 and Em 550 nm), injected via tail vein. Capillaries were stained with lectin. Adapted from Lee et al. (2012a). **(C)** Leukocytes present in the capillaries of an ischemic heart following reperfusion. **(D)** An image sequence shows sequential images of leukocytes (yellow) flowing through the capillaries (green). Red arrow heads indicate leukocytes transiting in the vessel. The average velocities of the leukocytes passing through the four capillaries indicated in **(E)** are plotted in **(F)**.

The technical progress described above has for the first time enabled real-time high resolution imaging of the mouse beating heart *in vivo*. It has also allowed acquisition of both structural and functional information at the cellular level during the cardiac cycle. In Figures 12–**15** we review some of our recent results in this field.

Typical examples of stabilized images of the cardiac microvasculature are shown in Figure 12. The in-frame motion artifacts, which were present within the original raw images have disappeared and motion-free images are reconstructed using a retrospective gating scheme in combination with SSM. By optically sectioning the cardiac tissue at different depths (here represented in 5-μm increments) *in vivo* 3D reconstructions of the beating heart are also easily obtained (Figure 12B; here the dimension of the 3D image is 256 × 256 × 75 μm in the x, y, and z directions, respectively, where the voxel size is 1 × 1 × 3 μm. Approximately 1000 beats were required to obtain this 3D reconstruction).

Thanks to the high degree of motion compensation achieved using these methodologies, high resolution imaging is feasible and both subcellular and subnuclear structures within the cardiac myocytes can be clearly resolved at different depths in the tissue (Figure 13).

Dynamic changes over the time scale of a cardiac cycle can also be visualized and quantified. As an example in Figures 14A,B, variations in a vessel diameter are reported at two distinct phases of the cardiac cycle. Furthermore, changes occurring longitudinally over a time frame of a few days can be measured. Large field of view images of a heart which sustained an ischemic reperfusion injury show migration after 24 h in the reperfused infarcted area of GFP-expressing cells, freshly isolated from the bone marrow of a donor mice (Figures 14C–F) and previously implanted in the heart's apex.

Motion compensation methodologies enable also *in vivo* measurements of dynamic cellular processes such as flow cytometry and cell tracking in the beating heart (Figure 15). Here stained leukocytes passing through a region of interest within a capillary are counted as a function of time. The average speed of leukocytes flowing through the capillaries can be likewise measured, making it possible to quantify changes in flow speed during the phases of the cardiac cycle or following reperfusion in infarcted hearts.

## Future perspectives

In this review we have reported on recent advances on innovative technologies aimed at reducing the motion-induced artifacts influencing microscopy imaging of the beating heart *in vivo*. The methods presented are based on advances in mechanical stabilization, gated and triggered acquisition protocols, and image processing algorithms. These methods do not affect the normal physiology of the heart and allow overcoming the motion-related limitations affecting cardiac intravital microscopy. In particular we have shown that the beating murine heart can be imaged at high temporal and spatial resolution as potentially offered by confocal and multiphoton microscopy, compensating for image distortion due to cardiac contraction and respiration.

We expect that the ability to interrogate the beating heart *in vivo* at physiological conditions and at the subcellular level will help to elucidate cardiomyocyte functions and interactions within the heart *in vivo*, with the ultimate goal of improving the treatment of cardiac diseases. Imaging and quantification of dynamic events in the myocardium including leukocyte trafficking, cell interactions, and drug pharmacology studies, will have a profound impact in expanding our current understanding of cardiac biology. Furthermore, intravital microscopy offers tremendous promise for the study of cardiac electrophysiology and arrhythmia at the single cell level. As described above, prospective gating with sequential segmented microscopy was recently demonstrated (Aguirre et al., 2014) and can enable motion-artifact free imaging of the beating heart at all phases of the cardiac cycle. This allows visualization and quantification of the single cardiomyocyte contractile cycle and can be readily expanded with fluorescent calcium and voltage reporters to measure dynamic contractile function. These methods will offer new tools for understanding excitation-contraction coupling in the heart and for mapping arrhythmia at the cellular scale.

In the coming years, many new developments are expected to further advance intravital microscopy for cardiovascular applications. These include the development of new passive and active stabilizer schemes as well as novel gating techniques and imaging protocols. With continued work on these methods and growing interest in the cardiovascular research community, intravital microscopy seems poised to impact the cardiovascular sciences in the way it has already propelled advances in the neurosciences and cancer biology.

### Conflict of interest statement

The authors declare that the research was conducted in the absence of any commercial or financial relationships that could be construed as a potential conflict of interest.
